# FK506 biosynthesis is regulated by two positive regulatory elements in *Streptomyces tsukubaensis*

**DOI:** 10.1186/1471-2180-12-238

**Published:** 2012-10-19

**Authors:** Dušan Goranovič, Marko Blažič, Vasilka Magdevska, Jaka Horvat, Enej Kuščer, Tomaž Polak, Javier Santos-Aberturas, Miriam Martínez-Castro, Carlos Barreiro, Peter Mrak, Gregor Kopitar, Gregor Kosec, Štefan Fujs, Juan F Martín, Hrvoje Petković

**Affiliations:** 1 Acies Bio d.o.o., Tehnološki Park 21, SI-1000, Ljubljana, Slovenia; 2 Department of Food Science and Technology, Biotechnical Faculty, University of Ljubljana, Jamnikarjeva 101, SI-1000, Ljubljana, Slovenia; 3 Instituto de Biotecnología de León (INBIOTEC), Parque Científico de León, Avenida. Real, No. 1, 24006, León, Spain; 4 Lek Pharmaceuticals d.d., a Sandoz company, Verovškova 57, SI-1526, Ljubljana, Slovenia; 5 Centre of Excellence for Integrated Approaches in Chemistry and Biology of Proteins, (CIPKeBiP), Jamova 39, Ljubljana, Slovenia

**Keywords:** FK506, Tacrolimus, *Streptomyces tsukubaensis*, Biosynthesis, Transcriptional regulator

## Abstract

**Background:**

FK506 (Tacrolimus) is an important immunosuppressant, produced by industrial biosynthetic processes using various *Streptomyces* species. Considering the complex structure of FK506, it is reasonable to expect complex regulatory networks controlling its biosynthesis. Regulatory elements, present in gene clusters can have a profound influence on the final yield of target product and can play an important role in development of industrial bioprocesses.

**Results:**

Three putative regulatory elements, namely *fkbR*, belonging to the LysR-type family, *fkbN*, a large ATP-binding regulator of the LuxR family (LAL-type) and *allN*, a homologue of AsnC family regulatory proteins, were identified in the FK506 gene cluster from *Streptomyces tsukubaensis* NRRL 18488, a progenitor of industrial strains used for production of FK506. Inactivation of *fkbN* caused a complete disruption of FK506 biosynthesis, while inactivation of *fkbR* resulted in about 80% reduction of FK506 yield. No functional role in the regulation of the FK506 gene cluster has been observed for the *allN* gene. Using RT-PCR and a reporter system based on a chalcone synthase *rppA*, we demonstrated, that in the wild type as well as in *fkbN*- and *fkbR*-inactivated strains, *fkbR* is transcribed in all stages of cultivation, even before the onset of FK506 production, whereas *fkbN* expression is initiated approximately with the initiation of FK506 production. Surprisingly, inactivation of *fkbN* (or *fkbR*) does not abolish the transcription of the genes in the FK506 gene cluster in general, but may reduce expression of some of the tested biosynthetic genes. Finally, introduction of a second copy of the *fkbR* or *fkbN* genes under the control of the strong ermE* promoter into the wild type strain resulted in 30% and 55% of yield improvement, respectively.

**Conclusions:**

Our results clearly demonstrate the positive regulatory role of *fkbR* and *fkbN* genes in FK506 biosynthesis in *S. tsukubaensis* NRRL 18488. We have shown that regulatory mechanisms can differ substantially from other, even apparently closely similar FK506-producing strains, reported in literature. Finally, we have demonstrated the potential of these genetically modified strains of *S. tsukubaensis* for improving the yield of fermentative processes for production of FK506.

## Background

FK506 (Tacrolimus) is a widely used immunosuppressant, produced by industrial fermentation processes using various *Streptomyces* species. Since its first clinical appearance in 1989 [1] it has been well established in medicine as an important immunosuppressant drug. The primary clinical utility of tacrolimus is prevention of graft rejection following organ and reconstructive tissue transplants and also treatment of skin diseases and eczema [2,3]. In recent clinical studies FK506-derived compounds have also shown promise for treatment of neurological disorders [4,5].

**Figure 1:**
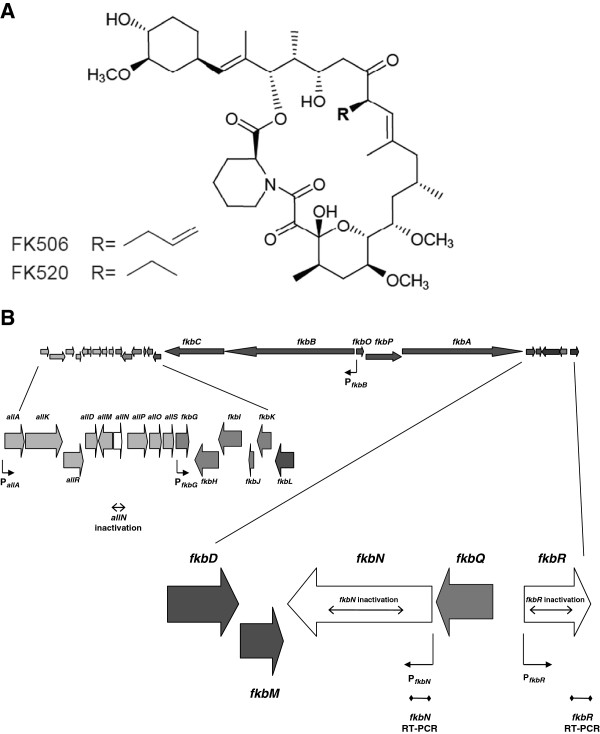
**(A) Structures of FK506 and FK520. **(**B**) Schematic representation of the FK506 biosynthetic cluster. The genes located on the left and right side from the FK506 core PKS region are presented in more detail. Putative regulatory gene homologues *allN*, *fkbN *and *fkbR *are represented by white arrows. Promoters used in the *rppA *reporter studies, deleted regions and RT-PCR amplified regions are marked.

A common feature of FK506 (Figure 1A), and its biogenetically and structurally related complex polyketides such as FK520 and rapamycin, is the involvement of large multifunctional polyketide synthase (PKS) / non-ribosomal peptide synthetase (NRPS) systems, comprising multi-fatty acid synthase-like domains arranged in sets of modules [6]. FK506 gene cluster from *Streptomyces* sp. MA6548 (ATCC53770) encoding the biosynthesis of this important drug was partially sequenced by Merck Research Laboratories [7-10]. In recent years, two entire gene clusters from *Streptomyces* sp. KCTC 11604BP and *Streptomyces kanamyceticus* KCTC 9225 [11], and a partial sequence of the FK506 gene cluster from *Streptomyces tsukubaensis* NRRL 18488 [12] have been published, thus allowing for the first time a comparative analysis of gene clusters involved in the formation of FK506 by different *Streptomyces* strains.

Better understanding of regulation of secondary metabolite biosynthesis could play a significant role in improvement of industrial strains, as has been exemplified in the past [13]. Regulation of secondary metabolism in actinomycetes is often diverse and complex and the production of active natural products is linked to many environmental and physiological signals [14]. In addition to numerous pleiotropic regulatory genes present in genomes of secondary metabolite-producing actinomycete strains, most of gene clusters encoding secondary metabolite biosynthesis contain pathway-specific regulatory genes, such as the SARP (*Streptomyces* antibiotic regulatory protein) family regulators [15] or the LAL (large ATP-binding regulators of the LuxR family) family regulators [16,17]. Like the SARP family, the LAL family gene-homologues with end-to-end similarity appear to be confined to the actinomycetes [18].

The production of many important polyketides or other secondary metabolites often remains relatively low and improving production titers of these low-yield compounds has been of great interest to the industry. This is particularly the case when considering FK506 and structurally related products, which are produced at rather low yields, compared to well established antibiotics of microbial origin such as penicillin, tetracyclines or polyether antibiotics [19]. Limited regulation aspects of rapamycin and FK520 biosynthesis have been studied in recent years [20-23]. Two regulatory genes, *rapH* and *rapG*, were identified in the rapamycin biosynthetic cluster and their role in regulation of rapamycin biosynthesis was confirmed [20]. Rapamycin RapH and its homologue in the FK520 biosynthetic cluster FkbN both belong to the LAL family of transcriptional regulators [16,24] since they both contain a LuxR-type helix-turn-helix (HTH) DNA binding motif at the C terminus [25] and an ATP-binding site at the N terminus [26]. In addition to *fkbN*, the gene cluster for FK520 biosynthesis from *Streptomyces hygroscopicus* var. *ascomyceticus* also contains a second regulatory gene, termed *fkbR1*, belonging to the LysR-type transcriptional regulators (LTTR) [21].

Until recently, regulatory genes have not been systematically investigated in FK506-producing strains. In the course of our recent work on FK506 biosynthesis [12,27] we have obtained a complete sequence of the FK506 biosynthetic cluster from *Streptomyces tsukubaensis* NRRL 18488. The obtained sequence allowed us to compare the putative regulatory elements present in our sequence with the other three FK506 gene clusters [11]. In addition, we have evaluated the role of three putative regulatory genes in the FK506 biosynthetic cluster using gene inactivation and over-expression approaches, as well as studied the transcription of FK506 biosynthetic genes in the mutant strains. In this work, we have demonstrated, that the biosynthesis of the FK506 in *Streptomyces tsukubaensis* NRRL 18488 is regulated by two positively-acting regulatory proteins, and remarkably, compared to the apparently closely-related strain, *Streptomyces* sp*.* KCTC 11604BP [28], it differs substantially.

## Methods

### Bacterial strains and culture conditions

We based our studies on *Streptomyces tsukubaensis* NRRL 18488 strain [12], a wild type progenitor of the industrially used FK506 high-producing strains. For spore stock preparation *S. tsukubaensis* strains were cultivated as a confluent lawn on the ISP4 agar sporulation medium [29] for 8–14 days at 28°C. For liquid cultures spores of *S. tsukubaensis* strains were inoculated in seed medium VG3 (0.25% (w/v) soy meal, 1% dextrin, 0.1% glucose, 0.5% yeast extract, 0.7% casein hydrolyzate, 0.02% K_2_HPO_4_, 0.05% NaCl, 0.0005% MnCl_2_ × 4H_2_O, 0.0025% FeSO_4_ × 7H_2_O, 0.0001% ZnSO_4_ × 7H_2_O, 0.0005% MgSO_4_ × 7H_2_O, 0.002% CaCl_2_, pH 7.0) and incubated at 28°C and 250 rpm for 24–48h. 10% (v/v) of the above seed culture was used for the inoculation of production medium PG3 (9% dextrin, 0.5% glucose, 1% soy meal, 1% soy peptone, 1% glycerol, 0.25%.

L-lysine, 0.1% K_2_HPO_4_, 0.15% CaCO_3_, 0.1% polyethylene glycol 1000, pH 6.5) [12,29]. Cultivation was carried out at 28°C, 250 rpm for 6–7 days. Kanamycin (25 μg/mL) and apramycin (50 μg/mL) were added to the solid and liquid media after sterilization as required.

### Recombinant DNA methods and bioinformatic analysis

Genomic DNA for sequencing and PCR amplification was prepared using standard procedures [30]. Plasmid vectors were propagated in *E. coli* DH10β grown in 2TY medium [31]. *S. tsukubaensis* transformation was carried out using *E. coli-Streptomyces* conjugation procedure with *E. coli* ET12567 containing the conjugation-facilitating plasmid pUZ8002 [32]. General *Streptomyces* strain manipulation was carried out using standard methods [30]. DNA manipulation was carried out using standard techniques [31]. Sequencing of the FK506 biosynthetic cluster of *S. tsukubaensis* NRRL 18488 strain was completed using 454 sequencing technology [33] at Macrogen, Inc., South Korea. DNA sequences covering the complete FK506 biosynthetic cluster and the right fringe of the FK506 gene cluster were deposited to the GenBank database with accession numbers [JX081655] and [JQ945188], respectively. Web-based versions of sequence database tools (BLAST programs at the NCBI server) and GC-content visualization (FramePlot program) were used for bioinformatic analyses [34-36]. ClustalW algorithm was used for DNA and protein sequence alignment [37].

### Overexpression of target regulatory genes in *S. tsukubaensis* strains

Primers for PCR amplification and cloning of the target putative *allN*, *fkbN* and *fkbR* genes (primers 1-6, see Additional file 1) were designed based on the newly acquired sequence of the *S. tsukubaensis* genome [12]. NdeI and XbaI restriction sites were incorporated via primers at the putative start codon and after the stop codon, respectively. PCR amplification was done using the Phusion® High-Fidelity DNA Polymerase (Fermentas). All PCR-generated fragments were purified using the Wizard® SV Gel and PCR Clean-Up System (Promega) after electrophoresis. The PCR fragments were initially cloned into pUC19 and their DNA sequence confirmed by sequencing. Further, the selected DNA fragments were excised from pUC19 using NdeI and XbaI restriction enzymes, gel purified and subcloned into the phiC31-based integrative expression vector pSET152, containing the constitutive ermE* promoter and a *Streptomyces* ribosome binding site [38], via NdeI and XbaI restriction sites, thus generating plasmids pDG1 (*allN*), pDG2 (*allN*+*mgl*), pDG3 (*fkbR*) and pDG4 (*fkbN*) (Table 1).

**Table 1:** Strains and plasmids used in this study

**Strain**	**Plasmid**	**Promoter**	**Gene**	**# if isolates**	**Description**
WT				11	Control
WT pSET152	pSET152			22	Control
WT *allN*	pDG1	P_ermE*_	*allN*	28	Over-expression
WT *allN*+*mgl*	pDG2	P_ermE*_	*allN*+*mgl*	25	Over-expression
WT *fkbR*	pDG3	P_ermE*_	*fkbR*	24	Over-expression
WT *fkbN*	pDG4	P_ermE*_	*fkbN*	29	Over-expression
Δ*allN*	pDG5		Δ*allN*	7	Gene inactivation
Δ*allN*Δ*fkbN*	pDG5, 8		Δ*allN *Δ*fkbN*	2	Gene inactivation
Δ*fkbN*	pDG8		Δ*fkbN*	10	Gene inactivation
Δ*fkbR*	pDG6		Δ*fkbR*	6	Gene inactivation
Δ*fkbR*Δ*fkbN*	pDG6, 8		Δ*fkbR *Δ*fkbN*	2	Gene inactivation
Δ*fkbR*	pDG3	P_ermE*_	*fkbR*	18	Complementation
Δ*fkbN*	pDG4	P_ermE*_	*fkbN*	33	Complementation
Δ*fkbR*Δ*fkbN*	pDG4	P_ermE*_	*fkbN*	9	Complementation
WT	pSET152		*rppA*	12	Negative control
Δ*fkbR*	pMB1	P_ermE*_	*rppA*	9	Positive control
	pMB2	P_*allA*_	*rppA*	15	Promoter activity
	pMB3	P_*fkbR*_	*rppA*	15	Promoter activity
Δ*fkbN*	pMB4	P_*fkbN*_	*rppA*	12	Promoter activity
	pMB5	P_*fkbB*_	*rppA*	15	Promoter activity
	pMB6	P_*fkbG*_	*rppA*	15	Promoter activity

By using a constitutive promoter ermE*, we reduced the potentially self-regulatory property of AsnC and LysR-type regulators, which are reported in the literature [39,40]. Constitutive transcription and relatively high strength of the ermE* promoter from *Saccharopolyspora erythraea* in the *S. tsukubaensis* NRRL 18488 strain was demonstrated previously in our work based on a reporter system, using the chalcone synthase *rppA* gene [41].

### Targeted gene disruption via homologous recombination

**Figure 2:**
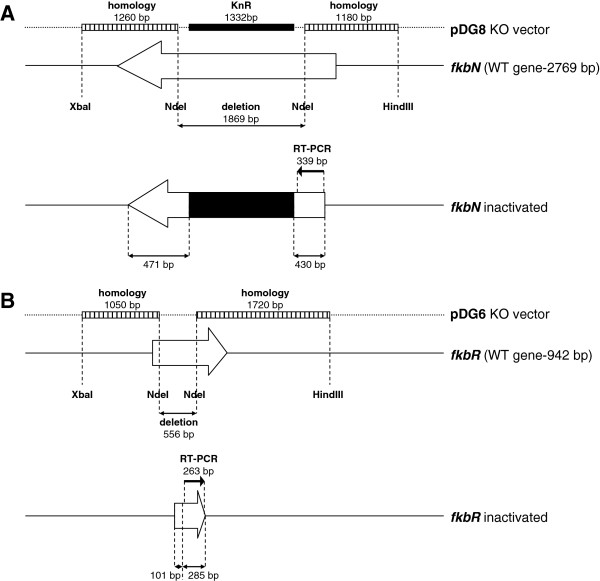
**Schematic representation of disruption plasmids and inactivated *fkbN *(A) and *fkbR *(B) genes after secondary recombination.**

We designed primers for amplification of the regions flanking the *allN*, *fkbR* and *fkbN* genes (primers 8-19, see Additional file 1). For the in-frame deletion of the *allN* gene, the upstream flanking region was amplified using primers containing EcoRI and XbaI sites and the downstream flanking region using primers containing XbaI and HindIII sites, thus generating a 292 bp in-frame gap in the 465 bp *allN* gene. For the disruption of *fkbR* the upstream flanking region was amplified using primers containing XbaI and NdeI sites and the downstream flanking region using primers containing NdeI and HindIII sites, thus generating a 556 bp in-frame gap in the 942 bp *fkbR* gene (Figure 2B; Additional file 2). For the disruption of *fkbN* the upstream flanking region was amplified using primers containing HindIII and NdeI sites and the downstream flanking region using primers containing NdeI and XbaI sites, thus generating a 1869 bp deletion in the 2769 bp *fkbN* gene (Figure 2A; Additional file 2). The PCR products were gel purified and ligated into the pUC19 vector and their nucleotide sequence was confirmed by sequencing. The DNA fragments were then excised from pUC19 using the corresponding restriction sites, that were introduced via primers, and gel purified. Both flanking regions were then subcloned simultaneously into the temperature-sensitive vector pKC1139 [42], containing a temperature-sensitive origin of replication in streptomycetes, which that was previously digested with corresponding restriction enzymes (EcoRI-HindIII for *allN*, XbaI-HindIII for *fkbR* and HindIII-XbaI for *fkbN* flanking regions), thus generating plasmids pDG5, pDG6 and pDG7 (progenitor of pDG8), respectively (Table 1). The primers for amplification of the regions flanking the target genes were specifically designed in order to create in-frame deletions after double cross-over recombination, thus avoiding the disruption of downstream genes due to polarity effect. In the case of the *fkbN* gene there was no need to ensure an in-frame deletion, because its coding sequence is located at the terminal position of the bicistronic mRNA and therefore the occurrence of a polar effect on downstream genes was not an issue (Figure 1B). Therefore, gene disruption procedure of the *fkbN* gene was aided by the introduction of a kanamycin resistance cassette in order to simplify the otherwise laborious identification of secondary recombinants. In order to introduce the kanamycin resistance cassette, the pDG7 plasmid containing the *fkbN* flanking regions was digested using NdeI, blunt-ended and dephosphorylated. A 1323 bp blunt-end fragment containing the kanamycin resistance cassette was excised from the SuperCos 1 cosmid vector (Stratagene) and then ligated into the vector, resulting in pDG8 (Table 1). The disruption plasmids pDG5, pDG6 and pDG8 were then introduced into electrocompetent *E. coli* strain ET12567 containing the conjugative plasmid pUZ8002 [32,43]. The conjugation procedure was carried out as described previously [42]. Exconjugants were grown at 28°C on ISP4 sporulation medium with addition of apramycin (pKC1139). Exconjugants were then inoculated into VG3 medium and cultivated at 28°C and 220 rpm to obtain a good seed culture [30]. After 24 hours, the cultures were reinoculated into a new tube with fresh VG3 medium and cultivated at 37°C. Above 34°C the pKC1139-based vector is unable to replicate and is forced to integrate into the *S. tsukubaensis* genome via homologous regions, thus yielding primary recombinants. The cultures were then further subcultivated at 37°C several times in VG3 medium and then plated onto the ISP4 sporulation medium. Harvested spores were filtered and serial dilutions were plated onto the sporulation medium without apramycin (with kanamycin in the case of *fkbN* disruption). After 5–8 days of cultivation at 28°C single colonies were replica-plated onto plates without antibiotic and plates with apramycin (both with kanamycin in the case of *fkbN*). Primary recombinants were still resistant to apramycin, while secondary recombinants lost apramycin resistance. The apramycin sensitive colonies were further screened using PCR to confirm the deletion. In the case of *fkbN*, the final screening step was simplified by the addition of kanamycin to the medium which precluded the growth of revertants to wild-type after secondary recombination, which greatly reduced the time and effort required to screen for correct secondary recombinants using PCR. After the stable secondary recombinants were identified and verified by PCR a double mutant was additionally generated in which both the *fkbR* and *fkbN* genes inactivated. Taking the *ΔfkbR* strain as the starting point we disrupted the *fkbN* gene using the same procedure as described above. Finally, all mutant strains were tested for FK506 production.

### Evaluation of the promoter activity of the selected genes from the *S. tsukubaensis* NRRL 18488 FK506 gene cluster using *rppA* reporter system

To study the promoter activity of selected genes from the FK506 gene cluster, which might be under control of FkbN and FkbR transcriptional regulators, we constructed reporter plasmids based on the chalcone synthase gene *rppA* from *Saccharopolyspora erythraea*, which was employed successfully in previous studies [20,41]. A series of plasmids were constructed containing the *rppA* gene as a reporter under the control of different promoters. Six putative promoter regions were selected; P_*allA*_, P_*fkbR*_, P_*fkbN*_, P_*fkbB*_, P_*fkbG*_, and P_ermE*_ (positive control), yielding plasmid constructs pMB1-6, representing different regions of the FK506 gene cluster (Table 1, Figure 1B). All promoter regions, except P_ermE*_, were PCR-amplified from *S. tsukubaensis* (NRRL 18488) genomic DNA. For PCR reactions primers were designed (primers 20-31, see Additional file 1) in a way to amplify approximately 500 bp of DNA upstream of the selected CDSs. PCR-amplified DNA fragments were gel-purified and ligated into the pUC19 vector. Their nucleotide sequence was confirmed by sequencing. The PCR-derived promoter fragments, containing EcoRI and NdeI sites were then fused at the NdeI site with the PCR-derived *rppA* gene, containing NdeI and XbaI and sub-cloned into pSET152 via EcoRI and XbaI sites. The “promoterless” *rppA* gene was also cloned into pSET152 and used in this experiment as a negative control. The plasmid constructs were then conjugated into *S. tsukubaensis* using *E. coli-Streptomyces* conjugation procedure as described earlier. Selected apramycin-resistant conjugants of *S. tsukubaensis* were cultivated in the PG3 production medium as described above until approximately 140 hours post inoculation. The culture broth was then centrifuged and the supernatant diluted 10 times and quantification of water-soluble dark-red flaviolin products of the chalcone synthase was carried out spectrophotometrically using the same conditions as described previously [41]. 270 nm was identified as the most appropriate wavelength for sample analysis and the expression of the *rppA* gene is presented as absorbance units (AU), taking into account the dilution factor. Thus, 1 AU represents the amount of flaviolin, which produces the difference in absorbance of 1 between the sample with an active promoter and the sample containing promoterless plasmid (blank) of the same strain at 270 nm (ΔA270).

### Gene expression analysis by reverse transcriptase PCR (RT-PCR)

In order to investigate further expression of regulatory genes and their influence on the expression of FK506-biosynthetic genes using a semi-quantitative RT-PCR approach, we have attempted to isolate good quality mRNA from cultures cultivated in the industrial production media (described above), but we were not successful. We therefore designed a simplified production media, which still contained the key ingredients from the industrial media. Simplified production medium SPM2 (6% soluble starch, 1% glucose, 0.2624% L-leucine, 0.25% L-lysine, 0.56% sodium lactate (60%), 1% MOPS, 0.05% NaCl, 0.05% MgSO_4_×7H_2_O, 0.0025% FeSO_4_×7H_2_O, 0.0005% MnCl_2_×4H_2_O, 0.001% ZnSO_4_×7H_2_O, 0.0003% CoCl_2_×6H_2_O, 0.0003% CuSO_4_×5H_2_O, pH 6.8) still gave a reasonable and relatively reproducible yield of around 20 mg/L of FK506 at the end of fermentation, as well as enabled good quality mRNA isolation. For the purpose of mRNA isolation, spores of *S. tsukubaensis* strains (1% v/v) were inoculated in the defined seed medium SVM2 (2% (w/v) soluble starch, 2% glucose, 2% yeast extract, 0.05% NaCl, 0.05% MgSO_4_×7H_2_O, 0.1% KNO_3_, 0.0025% FeSO_4_×7H_2_O, 0.0005% MnSO_4_×H_2_O, 0.001% ZnSO_4_×7H_2_O, 0.002% CaCl_2_×2H_2_O, pH 7.0) and incubated at 28°C and 220 rpm for 38 h. 10% (v/v) of the above seed culture was used for the inoculation of a 500-mL Erlenmeyer flask containing 100 mL of the production medium SPM2. Cultivation was carried out at 28°C, 220 rpm for 6–7 days.

For RNA extraction, 200 to 500 μL of culture (inverse proportion to the culture age) were added to 2 volumes of RNA Protect Bacteria Reagent (Qiagen), mixed by vortex (30 s) and kept 5 min at room temperature. The cell pellet was harvested by centrifugation (5 min, 10000 g), the supernatant was removed and samples were saved at -80°C.

Total RNA extraction method was based on that described by Tunca *et al*. [43]. The cell pellets were resuspended in 900 μL lysis solution [400 μL acid phenol, 100 μL CIA (chlorophorm:isoamyl alcohol; 24 :1), 400 μL RLT buffer (RNeasy mini kit; Qiagen)] and disrupted with a Fastprep instrument (BIO 101) by using the lysing matrix B (MP Biomedicals). Two pulses of 30 seconds and 6.5 of intensity were applied with cooling down for one minute on ice between pulses. Aqueous phase (containing RNA) was recovered after 10 minutes and 10000 g of centrifugation. Equal volume of CIA was added and the aqueous phase was again recovered after centrifugation (5 min, 10000 g). Subsequently, total RNA was isolated using an RNeasy mini kit (Qiagen) following the supplier’s indications. A second DNA removing step was carried out in solution using Ambion's TURBO DNA-free DNase. DNA contamination was tested for every set of primers (see Additional file 3) to confirm the absence of contaminating DNA in the RNA preparations. RNA concentration was calculated spectrophotometrically by determining the absorbance at 260 nm.

RT-PCR analysis was performed by using the SuperscriptTM One-Step RT-PCR with Platinum® Taq system (Invitrogen) with 50 ng of RNA as template and 35 cycles of amplification. Primers (see Additional file 3) were designed to generate PCR amplicons in the range of 200-500 bp and the annealing temperatures 55°C to 70 °C. Primer specificity was tested *in silico* by using the software available on the web site http://insilico.ehu.es[44]. Positive controls were done using as template total DNA of *S. tsukubaensis*.

### FK506 detection and quantification

Generally, at least nine independent exconjugants of each engineered strain were selected for further experiments. Data were obtained from at least two independent fermentation experiments. Extraction of FK506 and HPLC analysis were performed as described previously [12]. Briefly, after 6-7 days of cultivation the broth was mixed with the equal volume of methanol (1:1). Samples were filtrated and loaded onto Nucleosil EC100-3 C18, reversed-phase HPLC column. The mobile phase used for isocratic elution was composed of water, acetonitrile, MTBE and phosphoric acid (58.29:34.4:7.29:0.02, v/v/v/v). Chromatographic peaks corresponding to FK506 were identified and quantified using an FK506 external standard (obtained from Lek/Sandoz) and ChromQuest software was used for the data analysis. The calibration curve was generated using external standard prepared in the mobile phase and linear response was observed in concentration range from 1 to 1000 mg/L. Samples were analyzed immediately after each cultivation and for each experiment external standard was used for quantification. To obtain statistically significant results, each colony was represented by two parallel samples. Yields of FK506 were calculated with SAS/STAT software using means and the univariate procedure to test the normality of distribution. Using the GLM model, data were calculated as least mean square and are presented as an average change observed from all experiments when comparing least mean square values to the wild-type control least mean square value of each experiment.

## Results

### Bioinformatic analysis of the putative regulatory genes

Bioinformatic studies of the FK506 gene cluster from *Streptomyces tsukubaensis* NRRL 18488 revealed three potential regulatory genes; namely *fkbR*, *fkbN* and *allN* (Figure 1B). Two of the three putative regulatory genes, are located at the right side from the PKS core region, together with three coding sequences (CDSs) involved in biosynthetic reactions (Figure 1B), similarly to gene organization in the related FK506 biosynthetic cluster in *Streptomyces* sp. KCTC 11604BP [11]. The *fkbN* gene encodes a putative transcriptional regulator belonging to the LAL family [16,24] and *fkbR* encodes a putative transcriptional regulator belonging to the LTTR family and seems to represent the right limit of the FK506 gene cluster (Figure 1B). The product of the *fkbN* gene was originally typized by the regulator of the maltose regulon in *Escherichia coli* MalT [45]. These regulators are relatively large in size (872-1159 aa) compared to the better-studied SARPs (277-665 aa) [15] and they have been identified in several macrolide antibiotic pathways, including FkbN from *Streptomyces hygroscopicus* var. *ascomyceticus* in FK520 biosynthesis [21], PikD from *Streptomyces venezuelae* for pikromycin [46], RapH from *Streptomyces hygroscopicus* for rapamycin [20,24,47], NysRI/RIII from *Streptomyces noursei* for nystatin [48] and GdmRI and GdmRII from *Streptomyces hygroscopicus* 17997 for geldanamycin biosynthesis [49]. DNA sequence of the *fkbN* gene from *S. tsukubaensis* strain shares 69.9% amino acid identity (79.3% similarity) with FkbN from the FK520 cluster of *S. hygroscopicus* var. *ascomyceticus* and 57.4% amino acid identity (67.2% similarity) with RapH from the rapamycin cluster of *S. hygroscopicus*. The second regulatory gene, *fkbR*, displays all the usual characteristics of the LTTR family of transcriptional regulators; similar size (314 aa), a N-terminal HTH motif (residues 1-62) and the well conserved substrate-binding domains involved in co-inducer recognition and/or response [40,50,51]. Homologues of *fkbR,* the LTTRs, compose a family of autoregulatory transcriptional regulators that regulate very diverse genes and functions and are among the most common positive regulators in prokaryotes [40,51]. They generally do not exceed 325 aa residues in size, which was of great importance in assigning the correct start codon of *fkbR* in *S. tsukubaensis*. Further sequence analysis of the right fringe of the cluster suggests that an intergenic region of 430 bp seems to be present between the *fkbR* and thioesterase-encoding *fkbQ* genes, which are transcribed in opposite directions (Figure 1B).

In contrast to *fkbN* and *fkbR*, the third regulatory gene *allN* is located on the left fringe of the FK506 gene cluster where we have originally identified a number of CDSs involved in the provision of allylmalonyl-CoA [11,12]. The *allN* gene is a member of the AsnC family regulatory proteins, named after the asparagine synthetase activator from *E. coli*, which is known to be involved in the regulation of amino acid metabolism.

### Yield of FK506 is highly dependent on the expression of *fkbN* and *fkbR* regulatory genes

**Figure 3:**
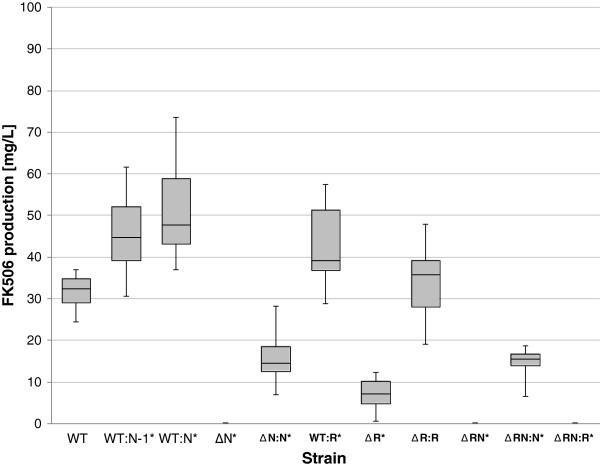
**Yield of FK506 by different strains of *S. tsukubaensis*. **Bars encompass 95% of the sample population. Horizontal line representing the median values, and perpendicular lines indicating extreme values (min, max). Asterisks where representing statistically significant differences between different samples compared to control wild type samples (WT). The data were analyzed using SAS/STAT program as described in Methods. Introduction of additional “in trans” copies of target putative regulatory genes using phiC31-based integrative vector [WT-wild type, WT:R-*fkbR *over-expressed, WT:N-1 (shorter version of *fkbN *over-expressed), WT:N-*fkbN* over-expressed, inactivation of target putative regulatory *S. tsukubaensis* genes (ΔR-*fkbR *inactivated, ΔN-*fkbN *inactivated) and complementation experiments (ΔR:R-*fkbR *mutant complemented with *fkbR*, ΔRN:N-*fkbR*, *fkbN *double mutant complemented only with *fkbN*, ΔN:N-*fkbN *mutant complemented with *fkbN*)].

In the next step our aim was to functionally characterize the three identified regulatory gene homologues in the FK506 biosynthetic cluster by gene-inactivation and overexpression experiments and to evaluate the possibilities for increasing FK506 yield by obtaining genetically engineered strains of *S. tsukubaensis*. It was not straightforward to identify the correct start codon for the CDS of the *fkbN* regulatory gene, since there are two possible start-codon sites located only 9 bp apart. We therefore amplified both versions of the gene, the longer *fkbN* and 9 bp shorter *fkbN-1* and carried out over-expression experiments using both PCR-amplified *fkbN* variants. The second copy of each version of the *fkbN* gene was introduced into the *S. tsukubaensis* wild type strain under the control of the strong ermE* promoter and *Streptomyces* ribosomal binding site (RBS) [38], a combination which was previously observed to enable high-level protein expression in this strain [41]. Overexpression of either version of *fkbN* resulted in improved FK506 production. In fact, the longer version of the *fkbN* gene proved to be more effective in increasing FK506 titers. An approximate raise in FK506 yield of 55% was observed in a growth medium that closely resembles industrial conditions when *fkbN* was introduced into the wild-type strain, while the shorter *fkbN-1* increased FK506 production by 38% (Figure 3). This demonstrates that the region surrounding the ATP-binding site at the N terminus of FkbN is important for complete functionality of the protein.

In contrast, inactivation of the *fkbN* gene caused complete disruption of FK506 biosynthesis (Figure 3), clearly demonstrating the key role of FkbN in the regulation of FK506 biosynthesis. When preparing the *fkbN* inactivated mutant (*ΔfkbN*) strain, a kanamycin resistance cassette was inserted into the *fkbN* CDS (Figure 2A). There was no need to ensure an in-frame deletion, considering that its coding sequence is located at the terminal position of the bicistronic mRNA and therefore a polar effect on neighboring genes was unlikely (Figure 1B). Finally, we have also carried out the complementation experiment with *fkbN* under the control of the constitutive ermE* promoter together with a *Streptomyces* RBS [38] in the Δ*fkbN* strains. After complementation FK506 production was only partially restored and reached 47% of the wild-type production. The Δ*fkbN* strains were complemented using the longer variant of the gene, which proved to be more effective in raising FK506 production in over-expression experiments. We have also complemented Δ*fkbR*Δ*fkbN*-double inactivated mutant strains. Interestingly, double “knock-out” mutants complemented with *fkbN*, reached comparable FK506 production levels (43%) to the Δ*fkbN* complemented strains (Figure 3). Therefore, although ermE* promoter (and heterologous RBS) is expressed strongly in *S. tsukubaensis*, as demonstrated previously by our group [41], it does not seem to be a suitable promoter to match “native” activity, which might require a specific mechanism of gene regulation, possibly also binding of a potential co-inducer. It is possible that the complementation experiment in the case of *fkbN* gene may have been influenced by the fact that although the transcription of *fkbN* gene was inactivated by deleting a big part of the CDS and inserting a kanamycin resistance cassette, the remaining parts of the gene, including the C-terminal DNA binding domain could still be expressed (see Additional file 2). Such truncated proteins could potentially interfere with the function of intact FkbN protein, produced in the complementation experiment. All this shows, that FkbN is indispensable for FK506 production, which is in agreement with recently published results [28]. Clearly, *fkbN* also shows important potential for application in genetic/metabolic engineering of industrial FK506 producing strains.

In the next step, an additional copy of the *fkbR* gene was introduced into *S. tsukubaensis* under the control of the ermE* and *Streptomyces* RBS [38]. Like in the case of *fkbN*, FK506-production was increased demonstrating that *fkbR* also has a positive regulatory role in *S. tsukubaensis* NRRL 18488. However, yield increase was moderate with FK506 production approximately 30% higher than in the control strain (Figure 3). The *fkbR* gene-disrupted mutants (Figure 2B; Additional file 2) displayed a significant reduction in FK506 production and on average they retained only approximately 20% of the wild-type production level, clearly demonstrating a positive role of this regulatory protein. Unlike FkbN, the FkbR regulatory protein is not indispensable for FK506-production. Interestingly, the Δ*fkbR* strains, complemented with the *fkbR* gene transcribed under the ermE* promoter showed recovery of FK506 production to wild-type levels (Figure 3). As expected, double mutant strains Δ*fkbR*Δ*fkbN* were unable to produce FK506.

Neither addition of a second copy of the *allN* gene transcribed under the ermE* promoter, nor the inactivation of *allN*, located on the left fringe of FK506 gene cluster, showed any influence on FK506 production or any other phenotypic characteristic (e.g. morphological), as the mutant strains retained wild-type values of FK506 yield. The result was the same when *allM* and *allN* were overexpressed together.

### Gene expression in FK506 gene cluster is not abolished by inactivation of *fkbN* or *fkbR*

In the next step we aimed to identify genes in the FK506 gene cluster, the transcription of which could possibly be regulated by FkbN and FkbR transcriptional regulators. We constructed reporter plasmids based on the *rppA* gene chalcone synthase from *S. erythraea*, described previously [20,41]. For the purpose of this work, we selected six different approximately 500-bp long putative promoter regions, located upstream of start codons of representative CDSs of the FK506 gene cluster. This allowed us to study the activity of putative promoters of predicted operons which encode different biosynthetic functions:

1) the promoter P_*allA*_ from *allA* gene located in the “all” subcluster (Figure 1B), which encodes a small diketide synthase system, part of a larger operon of four CDSs involved in the biosynthesis of allylmalonyl-CoA extender unit [12],

2) P_*fkbR*_ and P_*fkbN*_ promoters of CDSs encoding regulatory proteins FkbR and FkbN, respectively, which are located at the right fringe of the FK506 gene cluster,

3) P_*fkbB*_, a promoter of the largest CDS in the FK506 gene cluster encoding a PKS core region and

4) promoter P_*fkbG*_ of *fkbG*, a gene encoding a *O*-methyltransferase, located in the group of genes involved in the methoxymalonyl-ACP extender unit biosynthesis [21].

**Figure 4:**
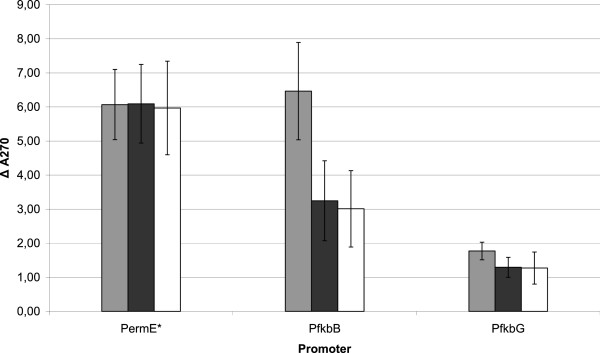
**Promoter activity represented as expression of the reporter gene *rppA *in *S. tsukubaensis *wild type and mutant strains (light gray – WT, dark gray – Δ*fkbR*, white – Δ*fkbN*). **The ΔA values represent the difference in absorbance at 270 nm, between the sample with an active promoter and the sample derived from the same mutant strain which was transformed by a promoterless plasmid (blank).

Promoter P_ermE*_, which was used as a control gave a strong consistent signal, confirmed by the constant expression of *rppA* in all tested *S. tsukubaensis* strains with engineered regulatory genes (Figure 4).

Wild type and *fkbN*- and *fkbR*-inactivated strains containing these plasmids were cultivated for approximately 140 hours whereupon the promoter activity of the cloned regions was assessed. Based on the *rppA* reporter, a significant change of expression was observed with P_*fkbB*_, the promoter of the gene encoding the largest coding sequence in the FK506 gene cluster, the first core PKS gene *fkbB*. In the wild-type strain, relatively high *rppA* reporter expression was observed under the control of P_*fkbB*_ promoter comparable to the control P_ermE*_ promoter, which is generally considered to be a strong *Streptomyces* promoter [52]. In the engineered mutant strains of *S. tsukubaensis* however, the identical construct containing the *rppA* gene under P_*fkbB*_, displayed significantly reduced production of colored flaviolin, 58% and 50% of the wild-type level for Δ*fkbR* and Δ*fkbN* inactivated strains, respectively (Figure 4). Interestingly, a complete loss of P_*fkbB*_ activity was not observed, even though FK506 production was completely abolished in Δ*fkbN* strains. In addition, we also observed a drop in activity of P_*fkbG*_ in both *fkbR* and *fkbN* inactivated strains. Although this experiment indicates, that expression of *fkbG* is at least partially regulated by FkbR and FkbN, relatively low signal and significant variations in absorbance among different independent strains were observed (Figure 4). Surprisingly, in all tested strains, in which the promoters P_*allA*_, P_*fkbR*_ and P_*fkbN*_ were tested, no differences in the OD_270nm_ values were observed, indicating very low levels of expression of the *rppA* reporter gene. This suggests a relatively low-level activity of these three promoters and, consequently, low level of expression of the genes encoding key steps in the substrate supply of the unusual extender unit, allylmalonyl-CoA, potentially influencing the ratio of undesired congener FK520.

**Figure 5:**
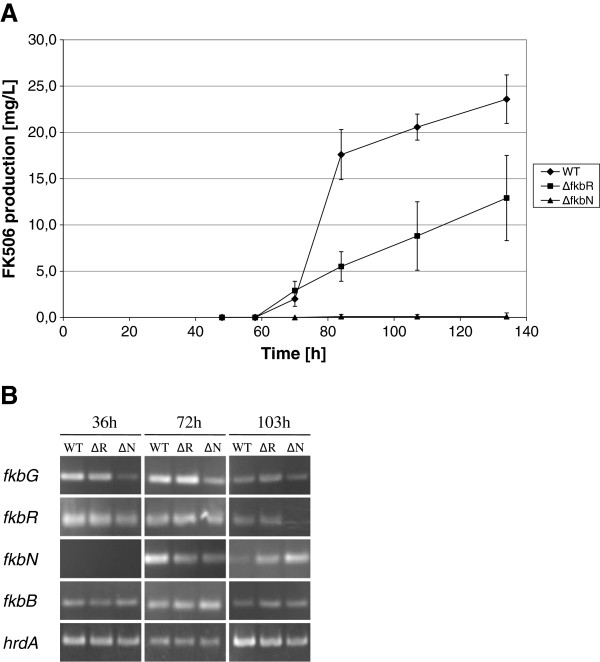
**(A) Time course for FK506 production in the SPM2 medium. **(**B**) Gene expression analysis by RT-PCR. Results of transcript analysis from three strains are presented WT-wild type, ΔR-*fkbR *inactivated, ΔN-*fkbN* inactivated. Total mycelial RNA was extracted after 36, 72 and 103 hours of fermentation.

In order to complement the interesting observations obtained by the chalcone synthase *rppA* gene-based reporter system, we also carried out semi-quantitative RT-PCR analysis of potential target genes of FkbN and FkbR (Figure 5B). Total RNA was isolated from the wild-type strain, *fkbR* and *fkbN* mutants after 36, 72 and 103 hours of growth in a modified liquid medium SPM2 (as described in Methods). We selected these intervals on the basis of FK506 production, which we followed in the same medium that was used for RNA preparation. FK506 production was first detected after approximately 50-60 hours and the production was highest around 70-80 hours of cultivation. After 103 hours of cultivation the culture was in the late stationary phase but was still producing FK506 at a moderate level (Figure 5A).

Interestingly, transcription of *fkbR* was observed already at early stage of cultivation (36 hours) and continued throughout the entire fermentation process. On the contrary, expression of *fkbN* was not observed in early stages of the fermentation process (before 36 hours) and was only detected around the onset of FK506 production (Figure 5B).

Surprisingly, inactivation of the *fkbN* gene, although completely abolishing FK506 biosynthesis, did not prevent the transcription of genes tested in the scope of this study. In agreement with results observed using the *rppA* reporter gene, we observed a decrease in transcription of *fkbG* (Figure 5B), the first of the five genes involved in the methoxymalonyl-ACP extender unit biosynthesis. However, FkbN protein is clearly not essential for transcription of *fkbG* as PCR bands can be clearly observed in the *fkbN*-inactivated strain as well as in the WT strain at early fermentation times when transcription of *fkbN* is still below detection limit of RT-PCR analysis (Figure 5B).

In contrast to the observations using the *rppA* reporter gene, where transcription of *fkbB* encoding the first part of FK506 PKS reduced significantly in *fkbN*-inactivated strain, RT-PCR approach did not show significant reduction of transcription of the *fkbB* gene.

Interestingly, in most RT-PCR experiments we were not able to detect any transcripts of the *allA* gene or in some cases, the corresponding RT-PCR bands were extremely weak, agreeing with the absence of any activity of chalcone synthase reporter *rppA* under the control of P_*allA*_. To re-confirm this result, we have designed more than one set of primers. The set of primers, which were used for RT-PCR experiments, resulted in successful amplification of the target PCR-product when DNA was used as template (data not shown). In conclusion, RT-PCR as well as *rppA* reporter gene approach showed that transcription of the tested FK506 biosynthetic genes is clearly not abolished upon inactivation of *fkbN* or *fkbR*. While both methods are essentially in agreement about this most important point, some discrepancies were observed regarding the changes in the level of expression for some of the tested genes, most remarkably the *fkbB* PKS gene. These discrepancies are further discussed below.

## Discussion

Biosynthesis of complex polyketides, such as biogenetically related immunosuppressants FK506 and rapamycin is likely tightly regulated, considering the complexity of the multienzyme machinery, which catalyzes the synthesis of such complex molecules. In this work, we have identified and characterized the functional role of two regulatory elements present in the FK506 biosynthetic cluster of *S. tsukubaensis* NRRL 18488 (Figure 1B). Our work, together with recent results of other groups demonstrates that regulatory mechanisms differ among different FK506 producing strains even though biosynthetic clusters appear to be very similar. Interestingly, two types of FK506 biosynthetic clusters seem to be present in different FK506 producing strains. The first group comprises FK506 gene clusters from *S. tsukubaensis* NRRL 18488 and *Streptomyces* sp*.* KCTC 11604BP with very similar nucleotide sequence and CDS-organization. These two gene clusters contain several additional CDSs, located in the “*all”* group of genes involved in biosynthesis of allylmalonyl-CoA extender unit, when comparing them to the second group of gene clusters from *Streptomyces tacrolimicus* (formerly *Streptomyces* sp*.* ATCC 55098 [53,54]) and *S. kanamyceticus* KCTC 9225 [11,12]. Gene clusters of all published FK506-producing strains contain an *fkbN* regulatory gene homologue, but only the larger version of gene clusters from *S. tsukubaensis* NRRL 18488 and *Streptomyces* sp*.* KCTC 11604BP contain another regulatory gene *fkbR* and an additional putative regulator *allN*[11].

Significantly lower yields of FK506 were generally observed in the *S. tacrolimicus* strain, containing the shorter version of the cluster (our unpublished results), therefore, the presence of additional biosynthetic and regulatory genes in the longer variant of the cluster might be related to better biosynthetic efficiency. Interestingly, it was reported that heterologous expression of *fkbR1,* a distant homologue of *fkbR* (49% nucleotide sequence identity, 24% amino acid sequence identity) from the FK520-producing strain *S. hygroscopicus* var*. ascomiceticus* in *S. tacrolimicus* resulted in a threefold increase of FK506 production [22,23]. Thus, it is reasonable to propose that at least one of the reasons for lower production by *S. tacrolimicus* strain could be the lack of *fkbR* regulatory element, in addition to the frameshift detected in the *fkbG* gene (hydroxymalonyl-ACP methyltransferase) [11]. In agreement with the findings of Won *et al*. [22,23] who observed positive effect of the heterologously expressed *fkbR1* gene in *S. tacrolimicus*, we have demonstrated that the native *fkbR* gene has an important role as a positive regulator of FK506 production in *S. tsukubaensis*. Overexpression of *fkbR* in the wild type *S. tsukubaensis* resulted in a relatively moderate increase in FK506 production, whereas its inactivation had a remarkable impact and decreased the production of FK506 to only a fifth of the wild-type level, clearly demonstrating its role as a positive regulatory element (Figure 3). In contrast to our findings, Mo *et al*. [28] have just recently reported that the *tcs7* gene (homologue of *fkbR*) from *Streptomyces* sp*.* KCTC 11604BP has a negative regulatory role. This seems to be a somehow surprising result considering extremely high degree of similarity of both FK506 biosynthetic clusters on the level of DNA sequence [11,28]. One possible explanation is that the two strains have different general (pleiotropic) regulatory networks and/or backgrounds of primary metabolic pathways, as has been observed recently in the case of allylmalonyl-CoA extender unit biosynthesis. In that case, the role of one of the FK506 biosynthetic genes (*allRtcsC*) was found to differ significantly in both strains in spite of identical nucleotide sequence of the gene. In *Streptomyces* sp*.* KCTC 11604BP this homologue of crotonyl-CoA carboxylase/reductase is involved exclusively in the biosynthesis of the allylmalonyl-CoA, an unusual building block of FK506 while on the other hand, in *S. tsukubaensis allR* also takes part in the biosynthesis of ethylmalonyl-CoA and thereby in the co-production of the FK520 impurity [11,27]. Comparative genomic analysis of these two strains should be carried out in the future in order to clarify the observed differences. Notably, in order to evaluate the potential of regulatory genes for increasing the yield of FK506 we carried out our experiments in media that closely resemble industrial conditions and therefore obtained considerably higher FK506 production. This may represent another explanation for the apparently divergent role of *fkbR/tcs7* in *S. tsukubaensis* NRRL 18488 and *Streptomyces* sp. KCTC 11604BP.

It was interesting to observe that when the *ΔfkbN* strain was complemented by overexpression of *fkbN* under the strong constitutive ermE* promoter, the FK506 production was not reestablished to its wild type levels. While the use of a heterologous constitutive ermE* promoter is one possible cause, another potential cause for only partial restoration of FK506 production of the complemented *ΔfkbN* strain may be that the *fkbN* gene was inactivated by replacing a central part of its CDS with a kanamycin resistance cassette. In this way, the N-terminal part of the CDS remains intact and may produce truncated proteins (Figure 2, Additional file 2). Such truncated fragments might potentially interfere with the normal function of intact FkbN proteins, expressed under the control of ermE* in the scope of the complementation experiment.

To evaluate the influence of *fkbN* and *fkbR* regulatory genes on the expression of FK506-biosynthetic genes, we carried out a transcriptional analysis of several selected genes using RT-PCR and, in parallel, the *rppA* chalcone synthase reporter system [20,41]. The main advantage of the *rppA* reporter is that it allows the use of nutrient-rich industrial media without any modification. Based on this reporter system, and in line with the hypothesis, that FkbR and FkbN are positive regulatory elements, we observed a decrease of expression of the PKS gene *fkbB* and possibly also of the methyl transferase gene *fkbG*, involved in biosynthesis of the methoxymalonyl-ACP extender unit in Δ*fkbR* and Δ*fkbN* mutant strains (Figure 4).

Considering that FK506 production was completely abolished in Δ*fkbN* strains, it is intriguing why the activity of P_*fkbB*_, was decreased in Δ*fkbR* and Δ*fkbN* strains to only approximately 58% and 50%, respectively, while a complete loss of the P_*fkbB*_ activity has not been observed. Interestingly, a very similar phenomenon was observed in the rapamycin gene cluster from *S. hygroscopicus* strain [20] and picromycin gene cluster from *Streptomyces venezuelae*[46]. These observations suggest that post-transcriptional regulation of polyketide biosynthesis may be an important and so far unexplored mechanism, possibly in part mediated by currently known regulatory proteins. It should be noted that a rare codon UUA is present in the *fkbN* transcript, providing an additional opportunity for translational regulation [55].

Further on, it is interesting to compare the results of *rppA* reporter gene experiments with the data obtained by RT-PCR experiments. Most importantly, both approaches are in good agreement that a general inactivation of transcription of all FK506 biosynthetic genes does not occur neither in Δ*fkbR* nor in the Δ*fkbN* strain, in which no FK506 is produced. In addition, both approaches showed a decrease of *fkbG* expression in the Δ*fkbN* strain (Figures 4 and 5B). This suggests that FkbN may positively regulate the expression of the genes involved in the methoxymalonyl-ACP extender unit biosynthesis at transcription level. On the other hand, it is intriguing to observe some degree of discrepancy between the two approaches, for example in the effect of FkbN and FkbR inactivation on *fkbB* expression. While *rppA* reporter system showed significant reduction of *fkbB* transcription (see above) the RT-PCR approach, in contrast, did not suggest any effect of *fkbN* inactivation on the transcription of this core PKS gene. Several reasons may account for the observed differences between the two approaches in levels of transcription of individual genes, for example: A) Flaviolin pigment, which is eventually produced by the *rppA* reporter gene, accumulates during the complete period of examination and can be seen as a “time accumulated” signal up to 140 hours when the samples were taken for analysis. On the other hand, RT-PCR provides snap-shot measurements of transcript levels. Considering that transcription of *fkbB* polyketide synthase gene is not completely abolished according to *rppA* reporter system, but only reduced to roughly 50% in the *fkbN*-inactivated mutants, time-dependent changes in levels of *fkbB* transcript may explain the observed divergence. B) Possibly, regulatory element(s) located outside of FK506 gene clusters in the two strains, might have a more prominent influence on regulation of the biosynthesis of FK506 than previously expected and may influence differently the P_*fkbB*_ promoter when located upstream of its native *fkbB* gene inside the FK506 cluster in contrast to when it is located in front of the *rppA* reporter gene in a different region of the chromosome. C) Similarly, different context of the P_*fkbB*_ promoter in *rppA* reporter system on one hand and in its native context on the other, may also give rise to different results in case truncated FkbN or FkbR proteins are expressed at low level as discussed above. Thus, our results show that the inactivation of *fkbN* nor *fkbR* had no significant general influence on the expression of most genes, located in the FK506 gene cluster, with the possible exception of *fkbG*, involved in the provision of methoxymalonyl-ACP. Although the used approaches enable only semi-quantitative assessment of differences in promoter activity our results suggest that the production of FK506 might in part be controlled by provision of this unusual extender unit. Obviously, this hypothesis will have to be explored in more detail in the future. Interestingly, recently published results by Chen *et al*. [56], seem to support this possibility as it was demonstrated that the over-expression of the methoxymalonyl-ACP providing genes under the strong constitutive promoter ermE* significantly increased the production of FK506 in *S. tsukubaensis*. In summary, we have clearly demonstrated, that inactivation of the *fkbN* gene, although completely abolishing FK506 biosynthesis, did not prevent the transcription of FK506 biosynthetic genes, contrary to the observations in *Streptomyces* sp*.* KCTC 11604BP strain, where all genes involved in biosynthesis of FK506 were silenced [28].

## Conclusions

Our results demonstrate that a complex regulatory mechanism is responsible for activation and complete functionality of the FK506 biosynthetic machinery. We show that, FkbN and FkbR clearly have a positive regulatory role in FK506 biosynthesis in the *S. tsukubaensis* strain when experiments are carried out in industrial-like fermentation medium. Remarkably, regulation of FK506 biosynthesis in *S. tsukubaensis* differs substantially from what has been recently described in *Streptomyces* sp. KCTC 11604BP [38] although the gene clusters of these two strains are practically identical on the DNA level. Most notably, we found *fkbR* to be a positively acting regulator in *S. tsukubaensis*, expressed continuously during the biosynthetic process. Moreover, the effect of *fkbN* inactivation on transcription levels of FK506 biosynthetic genes in *S. tsukubaensis* was not significant enough to account for complete disruption of FK506 production, suggesting that additional post-transcriptional regulatory mechanisms may be operative in this strain, while transcriptional regulation was found to be prevalent in *Streptomyces* sp. KCTC 11604BP. Significant differences in the regulation observed between these two strains obviously have a profound influence on the process development efforts at the industrial scale. Finally, we have demonstrated a potential for FK506 yield increase in engineered strains of *S. tsukubaensis* by simple overexpression of *fkbN* and *fkbR*, which could result in rapid and straightforward improvement of FK506 yield in the industrial fermentation process.

## Abbreviations

PKS: Polyketide synthase; NRPS: Non-ribosomal peptide synthetase; SARP: *Streptomyces *antibiotic regulatory protein; LAL: Large ATP-binding regulators of the LuxR family; HTH: Helix-turn-helix, LTTR, LysR-type transcriptional regulators; CDS: Coding sequence; WT: Wild type.

## Competing interests

The authors declare that they have no competing interests.

## Authors' contributions

DG and MB carried out cloning, overexpression and gene disruption experiments, promoter activity studies, bioinformatic and data analysis, participated in experiment design and drafted the manuscript. VM participated in the initial set-up of the chalcone synthase reporter system and provided support with the experiments. JH performed the HPLC and data analysis. EK participated in the design of the genetically manipulated strains. TP provided analytical support. JSA performed the RT-PCR studies. MMC and CB performed RNA isolation. PM and GKopitar provided support with gene cluster sequence analysis and experiment design. GKosec participated in the design of the study and manuscript correction. ŠF participated in experiment design and data analysis. JFM directed and supervised the RT-PCR experiments and corrected the manuscript. HP conceived and designed the study and corrected the manuscript. All authors read and approved the final manuscript.

## Supplementary Material

Additional file 1 Table containing primers for PCR amplifications of the target putative regulatory genes (The file presents primers and their corresponding sequences, that have been used for PCR amplification of whole genes or homologous regions and promoter regions). Click here for file

Additional file 2 Schematic representation of FkbR and FkbN protein domains and deleted regions (This file illustrates FkbR and FkbN proteins and their organization before and after inactivation). Click here for file

Additional file 3 Primers used for RT-PCR analysis (This file presents a list of primers and their corresponding sequences, that have been used for RT-PCR experiments). Click here for file
