# Supplementary material for: FK506 biosynthesis is regulated by two positive regulatory elements in Streptomyces tsukubaensis
Source: BMC Microbiol. 2012 Oct 19;12:238. doi: 10.1186/1471-2180-12-238 (PMC3551636; doi:10.1186/1471-2180-12-238)
Supplement: Additional file 1 — Table containing primers for PCR amplifications of the target putative regulatory genes (The file presents primers and their corresponding sequences, that have been used for PCR amplification of whole genes or homologous regions and promoter regions). [file 1471-2180-12-238-S1.pdf]

Additional file 1 Table of primers for PCR amplifications of the target putative regulatory genes

#	Primer name	Sequence	Restr. Site
1	AsnC-F	GCATATGAAAGAAAAGGTTGTCCTGGATTCG	NdeI
2	AsnC-R	GACCCCTGACTCCGGGCTAGTCTAGA	XbaI
3	fkbN-F1	CATATGGTTCCGGAAGTGCGAGCAGCCCC	NdeI
4	fkbN-F	CATATGCGAGCAGCCCCTTATGAACTGATCGC	NdeI
5	fkbN-R	GGGGCGACCGGTGCGGGTAGTCTAGA	XbaI
6	fkbR-F	GCATATGGAGTTACGCACCCTGGAGTACTTC	NdeI
7	fkbR-R	CAACCCGCCCCACAGGACTGAGAATCTAGAG	XbaI
8	AsnC-del-F1	GAATTCGGCGGCATCGGGTACACGCAC	EcoRI
9	AsnC-del-R1	GGCGTGGCACCGTCCACCTGTCTAGA	XbaI
10	AsnC-del-F2	TCTAGAGACCCACCTCGTATTCGGATCCTGG	XbaI
11	AsnC-del-R2	CCTGGACCGACTGCGCCCCAAGCTTC	HindIII
12	fkbR-del-F1	AAGCTTCGGGGTTACCAGGCGATGAC	HindIII
13	fkbR-del-R1	GAGGGCGATGTCGGCGGTCGCATATGG	NdeI
14	fkbR-del-F2	CATATGGCTGATCGCGGGCTGGACG	NdeI
15	fkbR-del-R2	GGAGCTGTGGCTGCGGCAGTTCTAGA	XbaI
16	fkbN-del-F1	CAAGCTTCTACCTTCCGATCGCCCGCG	HindIII
17	fkbN-del-R1	ACGTGCACCATGCCGACGGCGCATATGT	NdeI
18	fkbN-del-F2	CATATGCCCAAGCGGATCGACCTGCTC	NdeI
19	fkbN-del-R2	CCCGTTCGGCAGTCGCAGTGTCTAGAG	XbaI
20	rppA-F	ATGGAGGCACATATGGCAGTTCTATGCACCCCTGCG	NdeI
21	rppA-R	AATCTAGAGTCGGTCATCGGTTGCCTCCCGGGGCGGACGTG	XbaI
22	p_fkbR-F	TTTTGAATTCTTCGACGTTTTCTGCGGTCACCC	EcoRI
23	p_fkbR-R	TTTTTTTTCATATGGGCCGGGAGGCTAGCAACC	NdeI
24	p_fkbG-F	TTTCGAATTCGCGTTCTCGTGCTGACCAAGGTGTT	EcoRI
25	p_fkbG-R	TTTTTTTTCATATGGCCCGGAATGCTAGGCGGG	NdeI
26	p_fkbN-F	TTTTGAATTCCTGGACACGCCGATCACCGTATGT	EcoRI
27	p_fkbN-R	CTTTTTTTCATATGGGAGCACACGGCGCGA	NdeI
28	p_fkbB-F	AAAAAGGATCCCCGACACCGAACAGGTACTCGTC	BamHI
29	p_fkbB-R	CAAAAAAACATATGGAAAACGCCTTTCTCTCGGCTGAC	NdeI
30	p_allA-F	TTTTTGAATTCGAGTCCGCGGCACGGGCA	EcoRI
31	p_allA-R	CTTTTTTTTCATATGGTCATCGTCCTTTCGTCGGGA	NdeI
32	p_allA-F	TTTTTGAATTCGAGTCCGCGGCACGGGCA	EcoRI
