# Supplementary material for: FK506 biosynthesis is regulated by two positive regulatory elements in Streptomyces tsukubaensis
Source: BMC Microbiol. 2012 Oct 19;12:238. doi: 10.1186/1471-2180-12-238 (PMC3551636; doi:10.1186/1471-2180-12-238)
Supplement: Additional file 2 — Schematic representation of FkbR and FkbN protein domains and deleted regions (This file illustrates FkbR and FkbN proteins and their organization before and after inactivation). [file 1471-2180-12-238-S2.pdf]

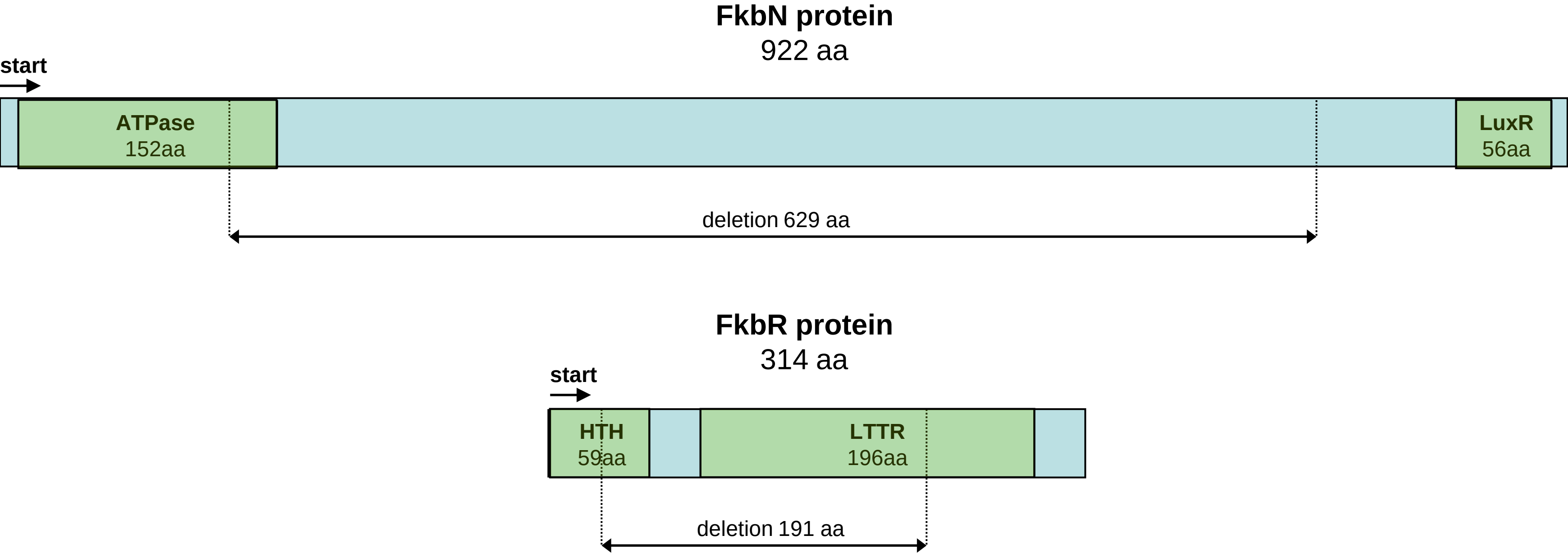

Additional file 3 Schematic representation of FkbR and FkbN protein domains and deleted regions
