# Supplementary material for: FK506 biosynthesis is regulated by two positive regulatory elements in Streptomyces tsukubaensis
Source: BMC Microbiol. 2012 Oct 19;12:238. doi: 10.1186/1471-2180-12-238 (PMC3551636; doi:10.1186/1471-2180-12-238)
Supplement: Additional file 3 — Primers used for RT-PCR analysis (This file presents a list of primers and their corresponding sequences, that have been used for RT-PCR experiments). [file 1471-2180-12-238-S3.pdf]

Additional file 2 Table of primers used for RT-PCR analysis

Primer name	Sequence	Amplicon length
fkbB-1_5	GCGGCGGCGTTGGCACCTCAT	280
fkbB-1_3	ACCCGCGCCCCGTCCTCCAAACTC	
*fkbG*_5	GTACGGATCGGCGACGCCCTGGAC	258
*fkbG*_3	GTCGCGCAGCCCCGCATTGAGTG	
*fkbN*_5	GGTGAACTCGGCCCGCAGC	339
*fkbN*_3	CGAGCAGCCCCTTATGAACTGATCGC	
allA-1_5	GCCCTGGCGGAGCTTCTGGAGTCA	283
allA-1_5	TCACCGTCTGGCTGGGGGAGTTCA	
allA-2_5	GGCGGGATGCTGGCGGTGAAGG	365
allA-2_3	CGGGTCATGTCGTTCACCATCAGG	
*fkbR*_5	GCCGACATCGCCCTCCAGGTG	257
*fkbR*_3	GGGTCCTGCGGGGCCTCTGC	
*hrdB*_5	GCCGCCGCGCCAAGAACC	419
*hrdB*_3	CCAGCGGCGTGTGCAGCGAGA	
